# Determinants, patterns, and gaps in patient safety and quality of care research in Israel: a scoping review

**DOI:** 10.1186/s13584-026-00749-0

**Published:** 2026-04-03

**Authors:** Merav Ben Natan

**Affiliations:** 1 Department of Nursing, Tel Aviv University, Tel Aviv, Israel; 2 Hillel Yaffe Academic School of Nursing, Hillel Yaffe Medical Center, P.O.B. 169, Hadera, 38100 Israel

**Keywords:** Patient safety, Quality of health care, Quality improvement, Health services research, Israel, Safety culture

## Abstract

**Background:**

Patient safety and healthcare quality are central priorities, yet preventable harm persists across health systems. Although Israel has implemented quality indicators, safety-culture initiatives and system-level reforms, the national evidence base has never been systematically reviewed. This scoping review aimed to map the extent, range and nature of empirical patient-safety and healthcare-quality research conducted in Israel between 2015 and 2025.

**Methods:**

The review followed JBI and PRISMA-ScR guidelines, with a protocol registered on the Open Science Framework. Systematic searches were conducted in MEDLINE, CINAHL, PubMed and Scopus to identify empirical studies examining patient safety or healthcare quality within the Israeli health system. Eligible studies were charted by design, setting, population, methods and thematic domain, and findings were synthesised narratively.

**Results:**

Twenty-eight studies met inclusion criteria, reflecting six thematic domains: organisational culture and reporting behaviour; medication and clinical-process safety; national quality-indicator performance; simulation and accreditation; ethics and patient rights; and crisis preparedness and resilience. Most studies were hospital-based and cross-sectional, drawing heavily on national indicators, structured safety-culture tools and simulation-based methods. Major gaps included limited research in community, mental-health, rehabilitation and long-term care settings, minimal use of interventional or longitudinal designs and scarce inclusion of patient or family perspectives. The evidence base remained predominantly focused on acute-care environments, with little attention to primary care or the wider care continuum.

**Conclusions:**

Israel has a growing but uneven published evidence base in patient safety and healthcare quality, characterised by strong hospital-focused research and comparatively limited representation of community and long-term care sectors in the peer-reviewed literature, even though national safety initiatives and monitoring frameworks operate across these settings. Advancing the field requires expanding research into under-examined settings, diversifying methodological approaches and more fully integrating patient perspectives. The findings provide an essential foundation for national improvement efforts and contribute to global discourse on patient-safety and quality advancement.

## Introduction

A commitment to improving the quality and safety of healthcare has been a central priority of governmental and organisational policy worldwide [1]. Despite these efforts, progress towards consistently safe care remains limited, with preventable harm continuing to affect patients across healthcare systems [2]. A key barrier to advancing patient safety is the limited availability of high-quality, comprehensive information that enables organisations and clinicians to assess performance and identify emerging risks. Such information is multidimensional, spanning clinical outcomes, process reliability, safety culture, and organisational learning systems, yet remains unevenly collected and applied [3]. Understanding how different healthcare systems approach these challenges is therefore essential for informing global learning and improvement efforts.

In Israel, quality and patient safety have become central to the Ministry of Health’s strategy. The Israeli National Programme for Quality Indicators (INPQ) introduced structured measurement and transparent reporting to strengthen accountability and organisational learning [4]. Despite this progress, the implementation and evaluation of safety interventions in Israel remain uneven, underscoring the need for a systematic analysis of how patient safety is enacted and investigated within the national healthcare system. In line with global trends, Israeli studies have increasingly examined safety culture, reporting behaviour, teamwork, medication safety, quality monitoring, simulation-based training, ethics, and emergency preparedness [e.g., 5–7]. However, these findings have not been synthesised to clarify national research activity, strengths, and evidence gaps.

Accordingly, this scoping review examines the extent, range, and nature of research on healthcare quality and patient safety conducted in Israel between 2015 and 2025. By mapping national research patterns, identifying dominant themes, and highlighting gaps, the review provides an evidence base to support improvements in practice, guide future research, and inform policy. The findings aim to contribute to the development of a more reliable, learning-oriented healthcare system and offer insights relevant to the international patient-safety community. To achieve these aims, this review addresses the following question: What is the extent, range, and nature of empirical patient-safety and healthcare-quality research conducted in Israel between 2015 and 2025?

## Methods

### Design

This scoping review was conducted in accordance with the PRISMA-ScR guidelines [8]. The primary review question examined the extent, range, and nature of research on healthcare quality and patient safety conducted in Israel between 2015 and 2025. The question was formulated using the PCC framework – Participants, Concept, and Context – which supported the definition of the review’s scope and informed the search strategy and study selection process [9]. The PCC elements were defined as: Participants (healthcare professionals, patients, or organisations in Israel), Concept (quality and patient safety), and Context (Israeli healthcare settings) (Table 1).

**Table 1:** PCC framework: definitions and search terms used in the scoping review

PCC component	Definition	Search terms
P – participants	Healthcare professionals, patients, or healthcare organisations involved in quality or patient-safety activities within Israel	“healthcare professionals” OR “patients” OR “healthcare organisations”
C – concept	Quality of care and patient safety, including safety culture, adverse events, reporting, medication safety, teamwork, simulation, accreditation, digital systems, and emergency preparedness	“patient safety” OR “quality of care” OR “safety culture” OR “adverse events” OR “medical errors” OR “reporting” OR “medication safety” OR “simulation” OR “accreditation” OR “emergency preparedness”
C – context	Israeli healthcare settings, including hospitals, community care, mental-health services, and long-term care facilities	“Israel” OR “Israeli healthcare” OR “Israeli hospitals” OR “Israeli health system”

### Eligibility criteria

Studies were eligible for inclusion if they were primary research articles published in peer-reviewed journals between 2015 and 2025, written in English, and conducted within the Israeli healthcare system or based on Israeli national datasets. Eligible studies addressed patient safety or quality of care, including topics such as safety culture, reporting behaviour, adverse events, medication safety, teamwork, simulation, accreditation, digital systems, and emergency preparedness. All major study designs were considered, including quantitative, qualitative, mixed-methods, simulation-based, and methodological validation studies. Studies were excluded if they did not focus on patient safety or quality, were not based on Israeli data, or were published as commentaries, editorials, conference abstracts, policy briefs, or dissertations.

### Search strategy

A comprehensive search of the literature was conducted across MEDLINE, CINAHL, PubMed and Scopus. Search terms were developed using the PCC framework and included combinations of keywords related to patient safety, quality of care, reporting, medication errors, safety culture, simulation, accreditation, and the Israeli healthcare system. Boolean operators, quotation marks, truncation, and parentheses were applied to maximize search sensitivity and precision. An example of the search structure included combinations such as: (“patient safety” OR “quality of care” OR “medical errors”) AND (“Israel” OR “Israeli healthcare”). Reference lists of all included articles were also reviewed to identify additional relevant studies.

Although the search strategy focused on patient safety and quality-related terminology, it is possible that some studies addressing national quality indicators (e.g., hip fracture outcomes or perioperative antibiotic use) were published without explicit reference to patient safety or quality concepts and were therefore not captured by the search terms alone.

### Study selection and data extraction

**Fig. 1:**
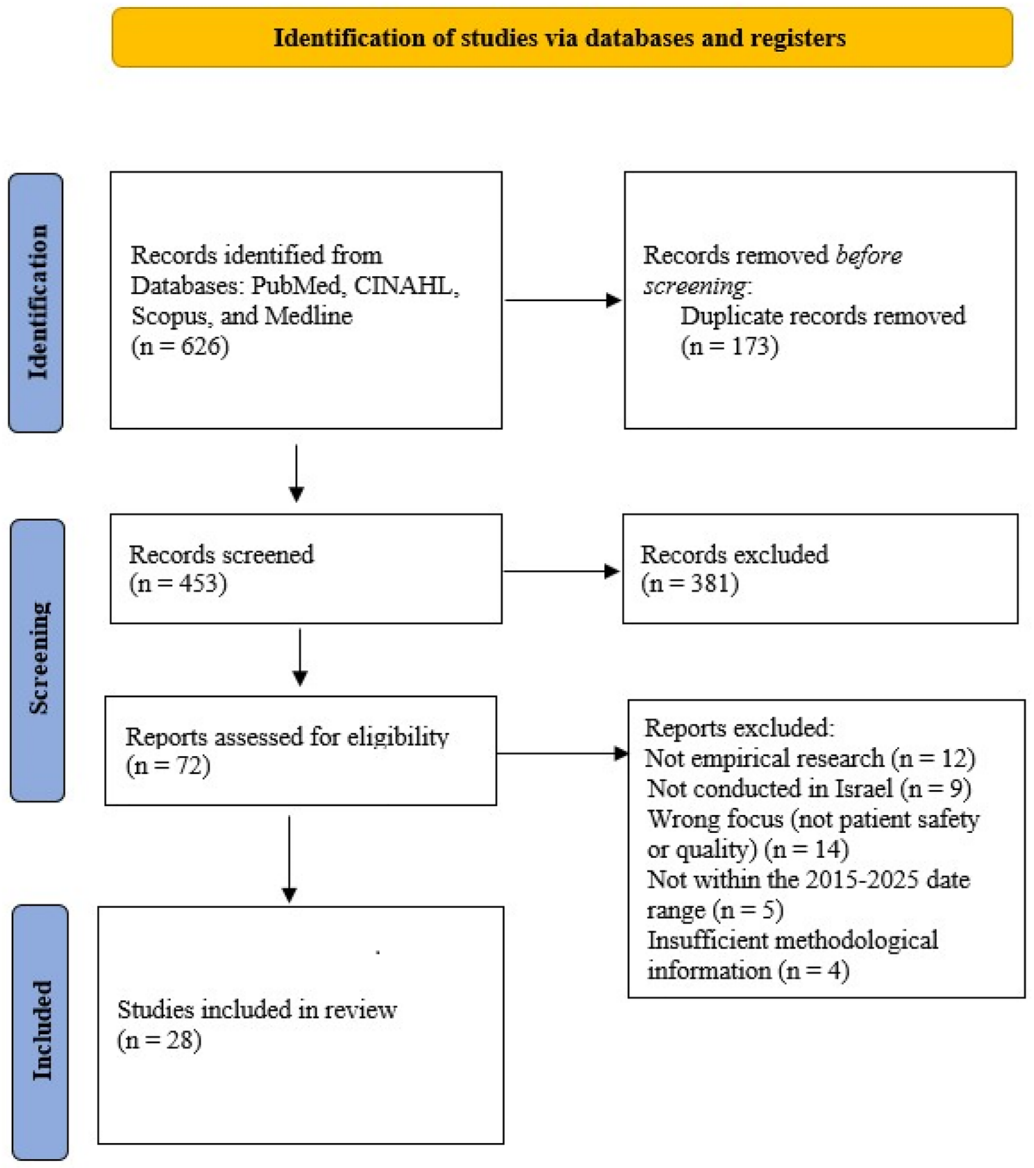
PRISMA flow diagram of study selection. Flow diagram describing the identification, screening, eligibility assessment and inclusion process of studies in the scoping review (2015–2025)

All records retrieved from the database searches were imported into EndNote and screened for duplicates. Titles and abstracts were then independently screened to assess relevance to patient safety and healthcare quality within the Israeli healthcare context. Records that clearly did not meet the inclusion criteria were excluded at this stage. Full texts of the remaining articles were subsequently retrieved and assessed for eligibility against the predefined inclusion and exclusion criteria. Screening and eligibility assessment were performed by the author and an independent reviewer with expertise in patient safety research. No artificial intelligence-based tools were used in the literature search, screening, or study selection. All stages were conducted manually according to the predefined protocol. Through this process, 28 studies met inclusion criteria and were selected for detailed analysis. The selection process is presented in a PRISMA-ScR flow diagram (Fig. 1). In alignment with scoping review methodology, no formal quality appraisal tool was applied. Data extraction was carried out using a structured extraction form and included information on study aims, design, setting, sample characteristics, instruments and variables, main findings, relevance to patient safety and quality of care, and reported limitations. Any uncertainties regarding eligibility were resolved through re-examination of the full text and discussion between the author and the independent reviewer.

### Analysis

A narrative thematic-synthesis approach was used to analyse and integrate the findings of the included studies [10]. Full texts were reviewed in detail to ensure a comprehensive understanding of each study’s context, methodology, and contributions. The analysis followed an inductive and iterative process, beginning with familiarisation with the data, followed by the development of initial descriptive codes. These categories were iteratively refined into overarching themes through repeated review and consolidation to ensure internal coherence and distinctiveness across themes. This process resulted in the identification of six major thematic domains that characterise patient-safety and quality-of-care research in Israel during the review period. The thematic-synthesis approach was selected for its suitability for integrating diverse study designs and for its capacity to map research patterns and highlight gaps in the evidence base.

## Results

The database search identified 626 records. After removal of 173 duplicates, 453 records were screened by title and abstract. Of these, 381 were excluded. Seventy-two full-text articles were assessed for eligibility, and 44 were excluded based on predefined criteria. Ultimately, 28 studies met the inclusion criteria and were included in the review (Fig. 1).

### Study settings, populations, and designs

The characteristics of the 28 included studies are presented in Table 2. (Table 2 about here) Of the 28 included studies, 20 (71%) were conducted primarily in hospital settings, including medical-surgical wards, intensive care units, paediatric emergency departments, operating rooms, internal medicine units, and trauma centres. Beyond hospital-based settings, national datasets were used to examine quality-indicator performance [5, 11], adverse-event trends [12], and hip-fracture surgical timing [13]. Other studies surveyed the general public [14], hospital staff [15], wartime physicians [16], and surgical inpatients [17], while one comparative OECD analysis positioned Israel within an international assessment of PROM/PREM development [18].

**Table 2:** Summary of the included studies

#	Authors	Year	Setting & population	Aim	Design	Key instrument	Main findings	Relevance to patient safety	Relevance to quality of care	Main limitations
1.	Niv et al. [5]	2025	National INPQ data	Indicator suspension	Retrospective	INPQ dataset	Performance sustained	Mature safety systems	Supports reallocating resources	No mortality data
2.	Toren et al. [6]	2022	17 hospitals	Evaluate ISBAR rollout	Mixed-methods	ISBAR survey	Communication improved	Standardised handoffs reduce errors	Improves continuity	Self-report; limited MDs
3.	Zimlichman et al. [7]	2018	4 hospitals	Validate ADE trigger tool	Retrospective review	IHI Trigger Tool	ADEs 7.5%; 22.7% preventable	Identifies hidden medication harm	Supports ADE-reduction programmes	Based on 2014 data
4.	Konson et al. [11]	2022	NPQI national dataset	COVID impact on indicators	Retrospective	NPQI indicators	Compliance stable or ↑; volumes ↓	Resilient safety structures	Maintained quality despite crisis	Admin data; no outcomes
5.	Arad et al. [12]	2024	National adverse events	Pre/during COVID	Retrospective	MOH event database	Adverse events ↑	Crisis vulnerability	Supports monitoring	Reporting bias possible
6.	Rozenfeld et al. [13]	2021	17 trauma centres	Early-surgery indicator & mortality	Retrospective	ITR registry	Indicator improved times; temporary mortality rise	Indicator changes affect safety	Guides careful rollout	Clalit-only data
7.	Kagan et al. [14]	2025	National general public	Transparency & safety	Cross-sectional	33-item tool	Low transparency	Highlights trust & communication	Improves engagement	Excludes Arabs; online bias
8.	Kagan, Porat & Barnoy [15]	2019	4 hospitals; patients + HCWs	Compare perceptions of safety culture	Cross-sectional	QPSC tools	Patients rated culture higher; predicts engagement	Shows patient feedback as safety indicator	Improves engagement & satisfaction	Self-report; variable survey versions
9.	Joachim et al. [16]	2024	3 hospitals	Resilience & danger	Cross-sectional	CD-RISC, danger scale	Higher danger near Gaza	Staff resilience essential	Supports mental-health policies	Cross-sectional
10.	Wolf et al. [17]	2025	General surgery ward	IH-MCI preparedness	Cross-sectional	MCI knowledge tool	75% unaware; mobility ↓	Patients vulnerable in disasters	Supports patient education	Single dept.; QR bias
11.	de Bienassis et al. [18]	2022	OECD incl. Israel	PROM/PREM harmonisation	Policy analysis	OECD PaRIS data	Few countries collect routine mental health PROMs	Patient voice enhances safety	Supports international benchmarking	No patient-level outcomes
12.	Toren et al. [19]	2021	3 hospitals; nurses	Predictors of near-miss reporting	Cross-sectional	HSOPS	High intention but low reporting; teamwork predicts reporting	Reveals intent–behaviour gap	Supports culture of feedback	Self-report; limited wards
13.	Naamneh & Bodas [20]	2024	Nurses	EMR effects	Cross-sectional	ENSS, EMR items	Errors ↓; workload ↓	EMR reduces errors	Improves documentation quality	Self-report
14.	Arad et al. [21]	2022	29 hospitals OR	Preop & intraop teamwork	Mixed-methods	Observations + interviews	Poor preop teamwork predicts poor intraop	Highlights checklist fidelity	Improves OR collaboration	Missing contextual data
15.	Finkelstein et al. [22]	2024	14 hospitals	Attitudes toward disclosure	Qualitative + survey	Workshops + pre/post	Strong support for disclosure	Supports transparent culture	Enhances trust & communication	Self-selection bias
16.	Semyonov-Tal [23]	2024	6 hospitals	Regulations & rights	Qualitative	Policy coding	Themes: autonomy, privacy	Rights frame safety	Improves patient-centred care	No implementation data
17.	Semyonov-Tal [24]	2024	6 hospitals	Regulations & rights	Qualitative	Policy coding	Themes: autonomy, privacy	Rights frame safety	Improves patient-centred care	No implementation data
18.	Sperling & Pikkel [25]	2020	National legal frameworks	Compare JCI rights vs Israeli law	Policy/legal review	5 acts + JCI PFR	JCI broader on access & culture	Rights-based care supports safety	Strengthens governance & consistency	No empirical validation
19.	Capua et al. [26]	2024	2 children’s hospitals	Room of Horrors	Pilot simulation	Hazard checklist	93% detection	Builds situational awareness	Builds team readiness	Small sample
20.	Walther et al. [27]	2022	Anaesthesia dept	Improve speaking up	Longitudinal QI	SAQ, SUPSQ	Psychological safety improved	Supports assertive communication	Enhances perioperative teamwork	Hawthorne effect
21.	Leviatan et al. [28]	2021	Sheba EMR dataset	Workload/shift effect on prescribing errors	Retrospective	MedAware AI	High workload & inexperience ↑ errors	Shows fatigue-related errors	Supports workload regulation	Single site; proxy measures
22.	Mekory et al. [29]	2017	Assaf Harofeh paediatrics	Errors before/during JCI	Retrospective	Chart review	Prescribing errors ↓; admin errors stable	Accreditation improves prescribing	Shows limits of non-digital systems	No long-term follow-up
23.	Susmallian et al. [30]	2022	Assuta ORs	Missing surgical items	Retrospective	Risk database	MSI rare; severe events ↓	Improves OR safety & counts	Strengthens teamwork & protocols	Single site
24.	Mendlovic et al. [31]	2023	Shaare Zedek	ED crowding intervention	QI (retrospective)	Operational data	Transfers ↑ but IM overload ↑	Local QI can shift risk	Shows ripple effects	Single site; no outcomes
25.	Gozlan et al. [32]	2024	Soroka; Oct 7 MCI	MCI response	Retrospective descriptive	Trauma & ops data	673 casualties; high resilience	Highlights MCI safety challenges	Supports preparedness	Single site; paper records
26.	Rappaport et al. [33]	2017	Meir MC; elderly patients	Medication reconciliation	Prospective	Drug lists (PCP vs RMU)	82% discrepancies; polypharmacy common	Shows high medication-risk burden	Supports structured reconciliation	Small sample
27.	Ein-Gal et al. [34]	2024	6 hospitals	Validate HSOPS 2.0	Psychometric	HSOPS 2.0	8-factor model	Enables national benchmarking	Enhances monitoring	Pilot only
28.	Ofek et al. [35]	2016	Assaf Harofeh	FMEA for KCl errors	FMEA	RPN scoring	Critical risks identified; 96% adherence post-policy	Removes high-alert KCl risks	Improves MMU safety	Single site; no long-term data

ADE = adverse drug event; AI = artificial intelligence; CD-RISC = Connor–Davidson Resilience Scale; COVID-19 = coronavirus disease 2019; ED = Emergency Department; EMR = electronic medical record; ENSS = Expanded Nursing Stress Scale; FMEA = Failure Mode and Effect Analysis; HCW = healthcare worker; HSOPS = Hospital Survey on Patient Safety Culture; IH-MCI = in-hospital mass casualty incident; IM = Internal Medicine; INPQ/NPQI = Israeli National Programme for Quality Indicators; IHI = Institute for Healthcare Improvement; ITR = Israel Trauma Registry; JCI = Joint Commission International; LOS = length of stay; MAE = medication administration error; MCI = mass casualty incident; MMU = Medication Management and Use (JCI standard); MSI = missing surgical item; OR = operating room; PaRIS = Patient-Reported Indicators Survey; PFR = Patient and Family Rights; PCP = primary care physician; PROM = patient-reported outcome measure; PREM = patient-reported experience measure; QI = quality improvement; QPSC = Quality and Patient Safety Culture; RMU = real medication use; RPN = risk priority number; SAQ = Safety Attitudes Questionnaire; SUPSQ = Speaking-Up About Patient Safety Questionnaire

Populations ranged from hospitalised adults, elderly patients, paediatric patients, and surgical inpatients to nurses, physicians, operating-room teams, hospital executives, and members of the general public [14–17]. A small number of studies focused exclusively on policy documents and regulatory frameworks [23–25].

Study designs included cross-sectional surveys [14–17, 19, 20], mixed-methods studies in operating rooms [21] and COVID-19 wards [22], and qualitative or documentary analyses of patient-rights legislation and hospital regulations [23–25]. Simulation studies assessed hazard detection and communication behaviours [26, 27]. Retrospective designs examined prescribing patterns [28], paediatric medication errors [29], adverse drug events using trigger tools [7], missing surgical items [30], quality-indicator performance [5, 11], adverse events during COVID-19 [12], emergency-department flow [31], and mass-casualty outcomes [32]. Prospective work included medication reconciliation [33]. Additional studies validated the Hebrew HSOPS 2.0 [34] and conducted a proactive FMEA on IV potassium chloride [35].

### Thematic domains identified

Six research themes emerged. Studies addressing safety culture, reporting behaviour, and workforce climate examined a range of perspectives across the healthcare system. These included public perceptions of safety [14], comparative assessments between patients and healthcare workers [15], nurses’ intentions to report near misses and adverse events [18], and teamwork dynamics in operating rooms [21]. Safety culture during the COVID-19 pandemic was explored among frontline nurses [22], while additional work focused on validating measurement tools such as the Hebrew HSOPS 2.0 [34], implementing structured speaking-up interventions [27], and examining attitudes towards error disclosure through multidisciplinary workshops [23].

The second domain, medication and clinical-process safety, included proactive and retrospective investigations across multiple stages of the medication-use process. These comprised analyses of high-alert medication handling using Failure Mode and Effects Analysis [35], medication reconciliation discrepancies among older adults [33], and the impact of accreditation on paediatric prescribing and administration errors [29]. Studies also identified workload-related risks in prescribing [28], documented adverse drug events via trigger tools [7], evaluated missing surgical items in the operating room [30], and examined the influence of teamwork and preoperative collaboration on intraoperative safety [21].

The third domain, performance monitoring and quality indicators, centred on national-level assessments. Analyses of the INPQ examined system performance during the COVID-19 pandemic [11] and the implications of suspending high-performing indicators [5]. Additional work assessed mortality trends following the introduction of early-surgery quality indicators for hip fractures [13].

The fourth domain, simulation, education, and accreditation, included studies evaluating structured educational strategies and accreditation processes. Simulation-based hazard detection was assessed in paediatric emergency departments [26], and speaking-up behaviours were strengthened through a longitudinal intervention in anesthesiology units [27]. Accreditation-driven improvements in paediatric medication safety were reported in [29], and the national implementation of the ISBAR communication protocol was evaluated in [6]. Accreditation was grouped within this domain because, in the included studies, it functioned primarily as a structured organisational and educational mechanism aimed at improving clinical processes and professional practice, rather than as a purely regulatory or rights-based framework. Although accreditation intersects with policy and patient-rights considerations, the empirical focus of the identified studies centred on its role in practice change, standardisation, and training-related implementation.

The fifth domain, ethics, policy, and patient rights, comprised policy-oriented analyses of regulatory frameworks. These studies examined the alignment between Israeli patient-rights legislation and accreditation standards [25], analysed hospital regulations governing autonomy, privacy, and consent [24], and evaluated the international development and harmonisation of patient-reported outcome and experience measures, including those relevant to Israel [18].

Finally, the sixth domain, crisis preparedness, mass-casualty response, and resilience, included research conducted during periods of national emergency. Adverse-event trends during the COVID-19 pandemic were examined in a national retrospective analysis [12], alongside analyses of quality-indicator performance under crisis pressures [11]. Studies also evaluated the unintended safety consequences of an emergency-department flow intervention [31], explored physicians’ resilience and perceived danger during armed conflict [16], documented the clinical and organisational response to the 7 October mass-casualty incident [32], and assessed hospitalised patients’ preparedness for internal mass-casualty events [17].

### Measurement tools and temporal patterns

Measurement tools included HSOPS/HSOPS 2.0, ISBAR surveys, EMR datasets, MedAware AI, medication reconciliation instruments, trigger tools, simulation checklists, resilience and danger scales, policy frameworks, risk-management databases, and national quality-indicator registries.

**Fig. 2:**
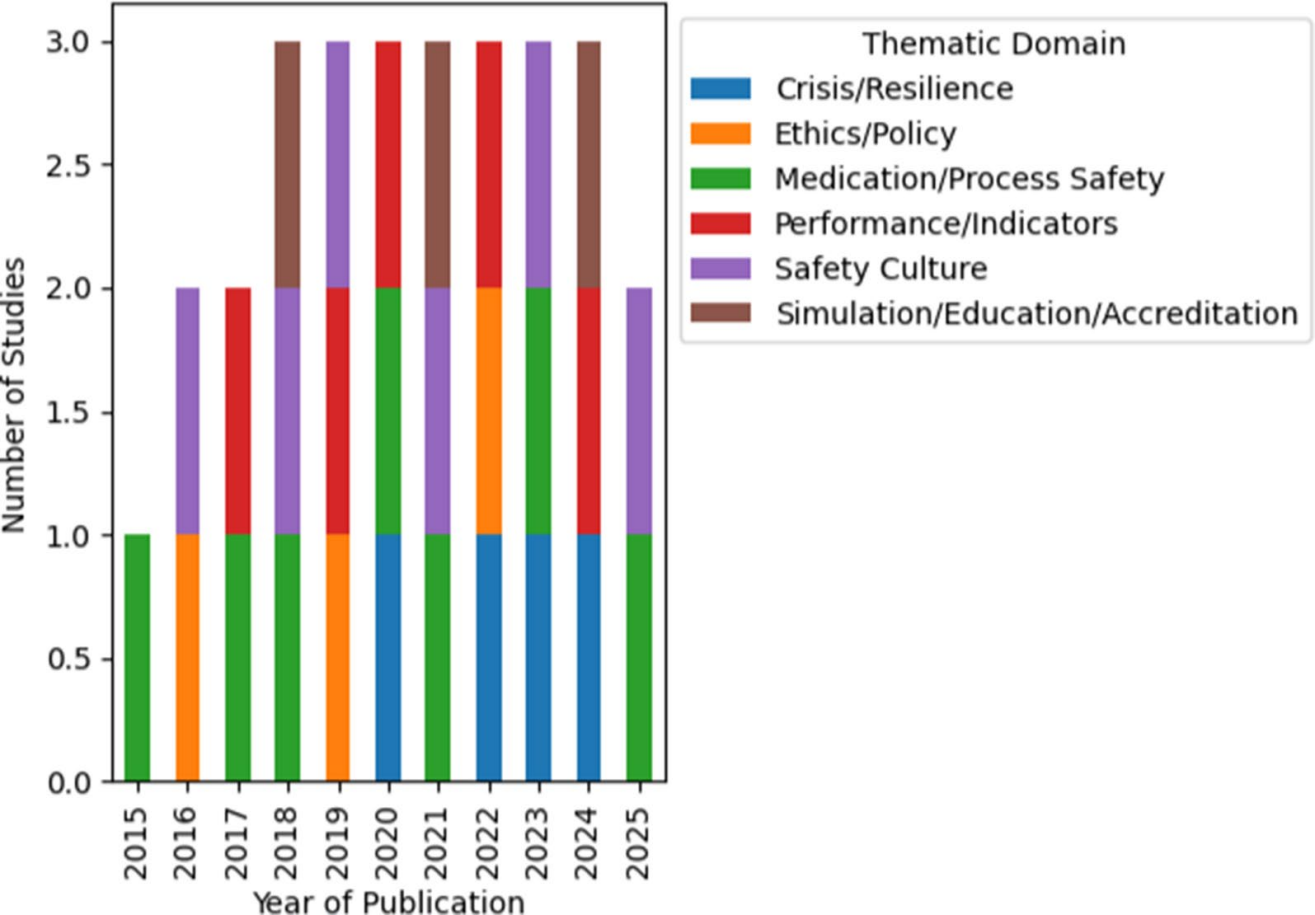
Distribution of studies by year and thematic domain (2015–2025). Bar chart presenting the number of included studies by year of publication and thematic domain

Publications spanned the entire decade, with a marked increase after 2018 and observable clusters aligned with major national events, including the COVID-19 pandemic and the 2023–2024 war (Fig. 2).

## Discussion

This scoping review synthesised 28 studies conducted in Israel between 2015 and 2025, revealing a diverse yet uneven body of research on patient safety and healthcare quality. Most studies were hospital-based and focused on safety culture, medication and clinical-process safety, and performance monitoring using national quality-indicator datasets. Additional work addressed simulation and accreditation, ethics and patient rights, and system resilience during emergencies including COVID-19 and the 2023–2024 war. Cross-sectional and retrospective designs predominated, while prospective, longitudinal, interventional, and community-based studies were less common. Across studies, the workforce perspective was central, whereas patient experiences and outcomes were examined far less frequently. Together, these findings indicate that patient-safety research in Israel is most developed in acute-care and organisational-performance contexts, with growing, but still limited, attention to equity, patient involvement, and preparedness for mass-casualty events.

An important consideration in interpreting these findings is that the mapped evidence reflects published research rather than the full scope of quality and patient safety activity within the Israeli healthcare system. Many quality and safety initiatives are implemented locally in response to accreditation and regulatory requirements and are not designed for scientific dissemination. Consequently, a substantial body of experiential and organisational knowledge remains outside the peer-reviewed literature. This observation is consistent with international evidence indicating that much quality improvement work is conducted as internal organisational activity rather than as formal research intended to generate generalisable knowledge [36, 38, 39]. The predominance of cross-sectional and retrospective designs and the limited number of interventional or multi-site studies identified in this review further suggest that many locally implemented initiatives are not systematically evaluated or translated into publishable evidence.

The findings of this review broadly align with international patient-safety research while also revealing several features distinctive to the Israeli context. Globally, safety-culture assessment, communication, teamwork, and medication-error prevention remain core priorities in safety science [36]. The concentration of Israeli studies in these areas mirrors international patterns, reinforcing their established role in shaping reporting behaviour, psychological safety, and clinical reliability [37]. At the same time, the strong national emphasis on quality-indicator monitoring, supported by large administrative datasets and long-standing Ministry of Health programmes, positions Israel alongside countries with mature performance-measurement infrastructures, such as the UK, Canada, and the Netherlands [38].

The widespread adoption of electronic medical records and national administrative databases has substantially expanded the capacity to conduct single- and multi-centre studies in patient safety and healthcare quality. These information systems enable linkage of clinical processes, outcomes, and organisational indicators across institutions and over time, thereby supporting more robust evaluation of safety initiatives and policy interventions. In the Israeli context, the availability of national quality-indicator datasets and digital health infrastructure provides a unique platform for longitudinal and comparative research. Leveraging these systems more systematically may facilitate a transition from predominantly cross-sectional studies towards interventional and multi-site designs capable of generating generalisable and policy-relevant evidence [38, 39].

However, this review also highlights gaps that echo global concerns. Several studies demonstrated that high performance on structural or process indicators does not necessarily translate into lower adverse-event rates, a trend observed internationally as healthcare systems encounter indicator fatigue and the limitations of metric-driven improvement [39]. The scarcity of community-based patient-safety research similarly reflects a recognised global blind spot, despite evidence that a substantial proportion of preventable harm occurs outside hospital settings [40].

An additional issue emerging from this review is the predominant focus of quality and safety measurement on process indicators rather than on patient-centred outcomes. Many of the included studies relied on indicators assessing adherence to clinical procedures, reporting behaviour, or organisational practices, while fewer examined clinical outcomes, functional status, or patient-reported outcomes. This pattern is consistent with international evidence showing that healthcare performance frameworks often prioritise process measures because they are easier to standardise and monitor, yet may have limited direct association with patient outcomes [39]. While process indicators play an essential role in supporting quality improvement and accountability, they do not fully capture the effectiveness of safety initiatives in improving patient health and wellbeing. Expanding the use and publication of outcome-based measures, including morbidity, functional recovery, and patient-reported outcome and experience measures (PROMs and PREMs), would provide a more comprehensive assessment of quality and safety interventions and enhance their policy relevance [43]. Greater integration of outcome indicators into national monitoring frameworks and peer-reviewed publications may also strengthen the clinical interpretability and international comparability of patient safety research [36, 39].

Crisis preparedness and mass-casualty resilience emerged as more prominent in the Israeli literature than in many high-income countries, reflecting the country’s unique geopolitical context. These findings parallel international evidence from healthcare systems exposed to war, natural disasters, and large-scale emergencies, which similarly report vulnerabilities in communication, reporting behaviour, and workforce capacity during crises [41, 42]. The Israeli studies therefore contribute valuable insights to the global evidence base on sustaining patient safety during prolonged operational strain.

This review also identifies an underdeveloped but internationally relevant area: the limited integration of patient and family perspectives in safety evaluation. Although interest in patient-reported outcome and experience measures is growing worldwide, routine implementation remains limited in many healthcare systems [43]. Israel’s participation in emerging international PROM/PREM harmonisation initiatives suggests improving alignment with global trends, but empirical evidence remains sparse.

Overall, the review not only maps national patterns but also contributes to the wider literature by offering a crisis-inclusive perspective on patient safety and healthcare quality within a highly stressed healthcare system. Evidence from studies conducted during the COVID-19 pandemic and periods of armed conflict indicates both system resilience, reflected in the maintenance of key quality indicators and adaptive organisational responses, and areas of strain, including increased adverse events, communication challenges, and workforce vulnerability under sustained operational pressure [11, 12, 16, 31, 32].

Findings indicate that while the Israeli National Quality Indicator Program encompasses community, pre-hospital care, geriatric, and psychiatric settings, the peer-reviewed literature remains heavily weighted towards hospital-based analyses. Published studies drawing on national indicator data from community, mental health, and long-term care sectors were limited within the scope of this review, suggesting a gap between existing measurement infrastructure and its representation in the scientific literature. Medication-safety risks identified in FMEA, trigger-tool analyses, and EMR-based studies were frequently examined within hospital settings, with reported associations involving decision-support tools, workload conditions, and reconciliation processes. Research addressing emergency and mass-casualty preparedness focused primarily on organisational response, workforce resilience, and operational performance during periods of crisis.

Future studies should broaden the scope beyond hospitals to include community clinics, home-care services, nursing homes, mental-health facilities, and rehabilitation centres. Prospective, longitudinal, and interventional designs are needed to assess the impact of safety initiatives over time. Research should incorporate patient perspectives more systematically, including PROMs, PREMs, and qualitative accounts of safety experiences. Finally, multidisciplinary research addressing equity, cultural competence, and accessibility is needed to ensure that safety strategies meet the needs of diverse populations within Israel and other multicultural healthcare systems.

Although the studies included in this review were published in established peer-reviewed journals in the fields of patient safety, quality of care, and health policy, the present scoping review did not conduct a bibliometric analysis of citation impact or journal impact factors. Such assessment falls outside the primary scope of scoping review methodology, which is intended to map thematic domains, study designs, and research gaps rather than to evaluate scientific influence. Future work could build on these findings by undertaking a dedicated bibliometric analysis to examine citation patterns, journal dissemination, and the extent to which methods and tools developed in Israeli patient safety and quality studies have been taken up in subsequent research and policy development.

### Policy implications and recommendations

The findings of this scoping review carry important implications for health policy and system-level governance in Israel. Although the national evidence base demonstrates substantial investment in hospital-based patient safety initiatives, the concentration of research within acute-care settings indicates a structural imbalance that may warrant policy attention. Expanding national patient safety strategies to encompass community clinics, mental health services, rehabilitation facilities, and long-term care institutions may help ensure that safety policies reflect the full continuum of care rather than hospital environments alone.

Strengthening the scientific contribution of quality and patient safety initiatives may benefit from embedding methodological rigor into project design from their inception, including clearly defined research questions, standardised outcome measures, and the use of longitudinal or interventional approaches where feasible. Early collaboration between healthcare organisations and academic institutions could enhance study design, data analysis, and publication readiness.

National leadership may play a central role in fostering such an infrastructure. A coordinated framework led by the Ministry of Health, in partnership with academic institutions, could support the transformation of institutional quality improvement projects into research initiatives with broader relevance. Competitive grant funding dedicated to patient safety and quality research may further incentivise high-quality project design and dissemination while ensuring alignment with national priorities.

Policymakers may build on existing national PROMs initiatives, such as those implemented for hip and knee replacement, by extending patient-reported outcome and experience measurement to additional clinical domains, including chronic disease management, mental health services, rehabilitation, and community-based care. Strengthening the routine publication and scientific dissemination of PROMs and PREMs findings may enhance transparency, support comparative evaluation across sectors, and contribute to a stronger evidence base for patient-centred policy development.

The review also indicates that reliance on performance indicators alone may be insufficient to achieve sustained improvements in patient safety. A policy shift towards organisational learning mechanisms, including structured communication tools, simulation-based training, and formalised error-disclosure programmes, may offer greater potential for long-term system improvement. These strategies are consistent with international evidence on the development of high-reliability healthcare organisations and the promotion of psychological safety among healthcare professionals.

Crisis preparedness and system resilience emerge as distinctive dimensions of the Israeli patient safety literature. Integrating patient safety principles explicitly into national emergency preparedness and disaster-response policies may help ensure that safety remains a core component of healthcare delivery under conditions of prolonged strain and may support continuity of care, workforce stability, and organisational adaptability during large-scale emergencies.

Finally, the findings highlight the importance of strengthening national research infrastructure to support longitudinal and interventional studies that evaluate the effectiveness of safety initiatives over time. Policy instruments that promote collaboration between academic institutions, healthcare organisations, and the Ministry of Health could facilitate the generation of actionable evidence to inform sustainable reform. Together, these policy directions underscore the central role of patient safety and healthcare quality within national health system governance in Israel.

## Limitations

This scoping review has several limitations that should be considered when interpreting the findings. A key consideration relates to the heterogeneity of the included studies in terms of design, scope, and methodological rigour. The predominance of cross-sectional and self-reported data limits causal interpretation and may introduce reporting or social desirability bias. However, given that the primary objective of this review was to map research activity rather than to evaluate intervention effectiveness, such heterogeneity reflects the breadth of the field rather than a methodological shortcoming of the review itself.

Another limitation concerns the concentration of peer-reviewed publications in hospital settings, resulting in comparatively limited representation of community, primary care, mental health, rehabilitation, and long-term care sectors. Although national quality-monitoring infrastructures encompass these domains, their underrepresentation in the published literature suggests a potential gap between measurement activity and academic dissemination. Consequently, the mapped evidence may not fully capture the scope of safety and quality initiatives occurring across the broader healthcare system.

The inclusion of English-language publications only may have led to the exclusion of relevant Hebrew-language studies or locally disseminated reports. In addition, the search strategy relied primarily on patient safety and quality-related terminology. Studies addressing national quality indicators or specific clinical domains may have been published without explicit reference to these conceptual terms and therefore may not have been retrieved. As a result, some relevant research activity may exist beyond the boundaries defined by the search framework.

Consistent with established scoping review methodology, no formal quality appraisal of included studies was conducted. Because the purpose of this review was to characterise the extent and nature of research rather than to synthesise effect sizes or determine evidence strength, methodological quality assessment was not undertaken. Nevertheless, the absence of formal appraisal limits conclusions regarding the robustness of individual studies.

Finally, the review included studies published up to 2025, and emerging developments may not yet be represented. Several included studies were conducted during crisis contexts, such as the COVID-19 pandemic or periods of armed conflict, which may affect the generalisability of findings to routine healthcare conditions.

## Conclusion

This scoping review offers the first comprehensive synthesis of patient-safety and healthcare-quality research conducted in Israel over the past decade. The evidence reveals a growing yet uneven published research landscape, characterised by strong hospital-based scholarship and comparatively limited representation of community, mental-health, rehabilitation, and long-term care sectors in the published literature, although national quality-monitoring systems extend across these domains. By mapping the dominant themes and identifying gaps, the review provides a clearer foundation for organisations and system leaders seeking to strengthen safety culture, enhance monitoring, and align improvement strategies with national and international expectations. The findings support practice by highlighting areas where structured communication, medication-safety processes, and performance measurement are well developed, and where additional investment, such as in community-based safety systems and patient-involvement mechanisms, is needed. Future research should diversify methodological approaches, include patient-reported measures, and expand beyond acute care to better represent the full care continuum.

Importantly, the prominence of crisis preparedness and mass-casualty resilience within the Israeli literature offers insights of broader international relevance. Evidence emerging from studies conducted during the COVID-19 pandemic and periods of armed conflict illustrates how healthcare systems can sustain safety monitoring, organisational adaptability, and workforce resilience under prolonged operational strain. These experiences may inform other countries seeking to strengthen patient safety frameworks in the context of large-scale emergencies and system-wide disruption. This work therefore supports international efforts to promote high reliability, transparency, and learning-oriented healthcare systems across both routine and crisis conditions.

## Data Availability

All data used in this study are derived from previously published sources. Extracted data and analytic materials are available from the corresponding author upon reasonable request.
